# Finding Ways to Lift Barriers to Care for Chronic Pain Patients: Outcomes of Using Internet-Based Self-Management Activities to Reduce Pain and Improve Quality of Life

**DOI:** 10.1155/2016/8714785

**Published:** 2016-03-01

**Authors:** Kevin Rod

**Affiliations:** ^1^Department of Family and Community Medicine, University of Toronto, 500 University Avenue, Toronto, ON, Canada M5G 1V7; ^2^Multi-Disciplinary Pain Management Centers, Toronto Poly Clinic, 5460 Yonge Street, Unit 204, Toronto, ON, Canada M2N 6K7

## Abstract

*Background*. Chronic pain is prevalent, disabling, costly, and undertreated. There is clearly a need to improve patient understanding of ways to manage their pain. Internet-based programs are continually being developed to facilitate mental health improvement, providing tailored content for patients to manage their pain, anxiety, and depression.* Objective*. To evaluate the impact of Internet-based patient self-management education and activities on patients' pain, anxiety, and quality of life in patients who could not access multidisciplinary pain management.* Design*. Observational study.* Subjects*. Two hundred (200) patients (61% females, 39% males, between 18 and 75 years old) from one community pain clinic in Toronto, Canada (Toronto Poly Clinic), participated. Patients had moderate to severe pain, depression, and anxiety. These patients committed to study from a group of 515 patients with chronic noncancer pain of different origins who were stable on their levels of pain, anxiety, and depression for 12 consecutive months before start of study and could not afford noninsured treatment modalities like physiotherapy, psychology, nutrition, or exercise therapy consultation.* Methods*. Patients were encouraged to visit two Internet sites (a blog and Twitter postings) for educational postings written by the author about exercise, nutrition, mindfulness meditation, disease management methods, evidence-based supplements, daily relaxation exercises, and overall self-management methods 15 minutes per day for six months. Patients were also encouraged to share their ideas and comments on a blog. Activity logs were kept by patients and reviewed by physician at follow-up visits. Compliance was encouraged via weekly email reminders and phone calls during the observation period.* Results*. Modest improvements were noted in pain, anxiety, depression, and quality of life. Of the patients with moderate or sever**e** pain before treatment, 45% reported mild levels of pain after treatment, with a reduction of severe pain from 40% before treatment to 25% after treatment (*p* value 0.0184).* Conclusion*. Self-management support interventions, such as Internet-based educational tools, can be considered to help patients manage their chronic pain, depression, and anxiety and may be helpful to improve the treatment outcome in patients who could not otherwise afford noninsured services.

## 1. Introduction

Chronic diseases, including chronic pain, affect a large portion of the population worldwide [1]. Although not life threatening, chronic pain can threaten quality of life from minor limitations to complete loss of independence [2]. Treatments for chronic pain are usually long-term and expensive and are mostly effective at managing rather than curing the condition. Pain is costly because it requires multiple medical treatments and complicates treatment for other ailments. Also, pain lowers worker productivity [3]. One study estimates that, in the United States (US) alone, the national cost of pain ranges from $560 to $635 billion, larger than the cost of the nation's priority health conditions and it suggests that because of its economic toll on society, the nation should invest in research, education, and training to advocate the successful treatment, management, and prevention of pain [3]. In Canada, a survey conducted in 2001 showed that the prevalence of chronic pain for adults older than 18 years of age was 18.9% [4].

Pain medicine experts agree that the successful management of chronic pain requires a multidisciplinary approach [5]. In an early study, the beneficial effects of multidisciplinary treatment were not limited to improvements in pain, mood, and interference but also extended to behavioral variables such as return to work or use of the health care system [5]. Many programs have shown outcome improvements by combining psychological, physical, and exercise therapies with proper medications, even reducing hospitalizations [6]. Although such programs are effective, patients often are limited in the amount of time and money they can allocate to such programs, making self-management support interventions important options to better manage their chronic diseases and improve treatment outcomes [7–10]. In addition, patients hoping for success with pain management programs often find themselves in a secondary struggle with health care coverage [11] that can be an unwelcome and harmful distraction to their battle with chronic pain. For example, many treatments may not be covered by health insurances or health insurance might not cover the physical therapy, but it will cover the medicine. Or if it covers physical therapy, it may only cover a few sessions. Many patients may not have private insurances, and the cost of treatment becomes a major barrier for them to access proper care.

In this observational study, we used an Internet patient education blog and Twitter postings to provide free, accessible patient self-management education for a group of chronic pain patients and evaluate the impact of these tools on patients' levels of pain, anxiety, depression, and quality of life. The goal was to observe if using Internet-based patient self-management education would improve measurable outcomes in chronic noncancer patients who were stable on their severity of pain, depression, anxiety, and impact on quality of life for one year and were not able to access noninsured services otherwise.

## 2. Methods

### 2.1. Patients

As a community chronic pain management center, this clinic treats a large group of patients who try many different treatment modalities alone or combined in different treatment plans for chronic noncancer pain. Some of these treatment modalities are not covered by their provincial health insurance plan and this becomes a barrier to care for patient who cannot afford them. This can impact their treatment outcomes. We measure the levels of pain and comorbid conditions of depression and anxiety along with impact on quality of life through standardized questionnaires (Numeric Pain Rating Scale, Hamilton Depression Rating Scale, Hospital Anxiety and Depression Scale, Quality of life Scale, and for posttreatments follow-up visits the Patient Global Impression of Change Scale) for all patients before treatment and in follow-up visits to monitor our treatment outcomes. Many of these patients do not have access to noninsured treatment services like physiotherapy, psychology, nutrition, or exercise therapy consultations. We identified a group of 515 of this group of patients who despite trying all treatments available to them through insured services (medications, limited counselling, and nerve block or trigger points injections) had significant pain (moderate or severe pain), anxiety, depression, and poor quality of life. These patients had plateau levels of their pain and limitations for at least 12 months. These patients had no prior training or education in self-management techniques. This group of patients were provided with the opportunity to participate in this observation for six months. For inclusion 18 to 75 years old patients who had at least moderate levels of pain, depression, and anxiety with quality of life level of less than five for a period of at least twelve months and had no plan or financial possibility of changing any of their concurrent treatments for the period of study (six months) were considered. Patient who had plans to add noninsured services like physiotherapy, psychological therapy, nutrition, and exercise therapy consultation to their current treatments were excluded. Patients with concurrent conditions of cancer, psychosis, unstable bipolar affective disorder, lack of proper command of English language, and inability to access or use Internet for education and patients with possibility of being scheduled for any surgery were excluded. Ethics board approval was obtained for this observational study on effects of online patient self-management education. Patients of this group were provided with possibility of and education about participation in this study and the informed consent process. We anticipated having at least 15% improvement in different measurable scales of pain, depression, anxiety, and quality of life.

Two hundred (200) chronic pain patients (61% females, 39% males) agreed to participate in this 6-month study (from the fourth quarter of 2014 to the first quarter of 2015). The main reasons for declining to take part in the study were lack of consistent access to Internet, not being familiar enough with Internet, or not being able to commit to regular daily 15 minutes' activities. Patients had access to a patient educational blog (http://mypain.ca/) and Twitter postings under the title #ZENDOSE (https://twitter.com/search?q=zendose&src=typd/) to learn how to be safely active, improve eating habits, manage stress, learn mindfulness skills, and review proper medication and supplement use. The online materials were provided by the author based on the current evidence-based literature in the related fields. Patients were encouraged to read the educational postings (http://mypain.ca/) about exercise, nutrition, meditation, disease management methods, evidence-based supplements, daily relaxation exercises, and overall self-management methods and the mindfulness postings on Twitter under #ZENDOSE and reflect on the sites materials for a minimum of 15 minutes a day. Patients were encouraged to share ideas and comments on the blog by logging in with their Facebook, Google, or Twitter accounts. The #ZENDOSE posts were updated daily, and patients were encouraged to reflect on the messages for 15 minutes each day. No other elements of their treatments (medications and limited counselling and injections) were changed, and patients were still following up with their usual clinic visits. To encourage compliance, email and phone reminders were made on a weekly basis. Patients kept logs to record time spent on the sites to assure a minimum of 15 minutes of reading and reflection. Patient logs were reviewed by treating physician on their regular follow-up visits. We noticed marked changes as described in the study results. The 315 patients who did not participate continued to have the same plateau level on all levels of outcome measurements through this six months' period of time.

### 2.2. Procedure

In patients with chronic pain, pain, depression, anxiety, and poor quality of life are significant issues that affect different aspects of their lives. The Numeric Pain Rating Scale (NPRS), Hamilton Depression Rating Scale (HDRS), Hospital Anxiety and Depression Scale (HADS), and Quality of Life Scale (QOLS) have been previously used in different research studies, and their validity as research scale tools has been demonstrated [12–15]. We used these standardized scales to measure pain, anxiety, depression, and quality of life in this chronic pain patient population.

All patients who were enrolled had at least a moderate level of pain on the NPRS (0 = none; 1–3 = mild; 4–6 = moderate; and 7–10 = severe) [16]; at least a moderate level of depression on the HDRS (0–7 = normal, 8–13 = mild depression, 14–18 = moderate depression, and 19-20 = severe depression) [17]; at least moderate anxiety on the HADS 8–10 = borderline anxiety, 11–21 = abnormal anxiety and depression [18]; and less than 5 with regard to the inability to function in daily activities (0 = nonfunctioning, 10 = normal) on the QOLS [16]. Patients were evaluated at baseline, during their follow-up visit, and at end point. At the end of the study, patients completed the Patient Global Impression of Change (PGIC) [19, 20]. Baseline pretreatment measures and end of six months' measures were compared.

Compliance and support emails were sent to all patients on a weekly basis during the six-month observation period. Patient compliance logs were reviewed by treating physician on regular follow-up visits. All other elements of treatment were kept the same as baseline without any changes during the observation.

### 2.3. Statistical Analysis

A sample size of 200 patients achieves 84% power to detect a difference of 0.10 after treatment using a 2-sided binomial test with an alpha = 0.05. This assumes that the pretreatment population proportion under the null hypothesis is 0.40.

The proportion of patients with moderate or severe pain, depression, and anxiety was calculated by taking the number of subjects scoring in the respective categories using the standard questionnaires and dividing by 200 patients. The proportion of patients with the ability to function in daily activities before and after treatment was also calculated. The change in proportion of subjects from the period before to the period after treatment was reported as the improvement.

The significance level of the improvement in pain, depression, and anxiety was assessed using a general *z*-test [21] to test the null hypothesis that the improvement is equal to 10%. Patients who had severe pain before treatment were assessed separately. Then patients who had moderate or severe pain before treatment were also assessed.

The proportion of patients with a global impression of change after treatment was also calculated.

## 3. Results

**Figure 1:**
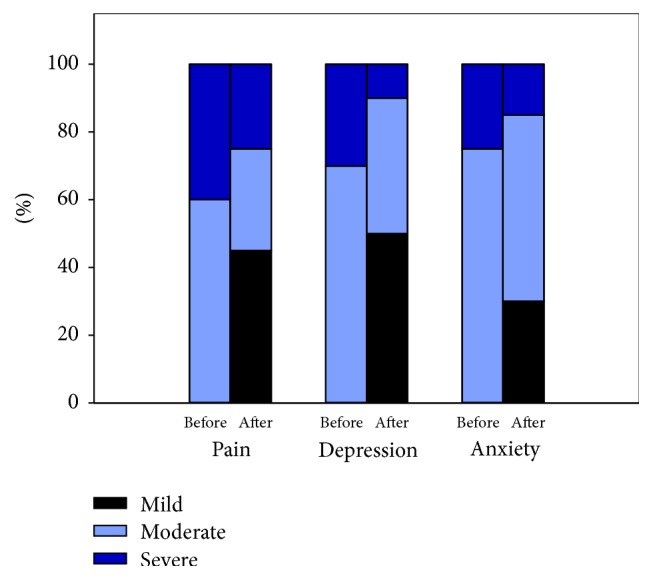
Perception of severe pain depression and anxiety.

Modest improvements were noted in pain, anxiety, depression, and quality of life. Of the patients with moderate or sever**e** pain before treatment, 45% reported mild levels of pain after treatment, with a reduction of severe pain from 40% before treatment to 25% after treatment (Figure 1).

On the depression scale, severe depression was observed to be reduced from 30% before treatment to 10% after treatment and, of all of the patients who participated in the study having moderate or severe depression before treatment, 50% reported mild depression after treatment. On the anxiety scale, the severe anxiety group was reduced from 25% before treatment to 15% after treatment. Quality of life improved from 25% before treatment to 60% after. More than half (60%) of patients reported much improved results on the PGIC scale after treatment (Table 1).

## 4. Discussion

Chronic pain comes at a cost whether from lost wages, social stigma, or ineffective health care coverage. One study found that the annual cost of pain was greater than the annual costs of heart disease ($309 billion), cancer ($243 billion), and diabetes ($188 billion) [3]. Despite the success of multidisciplinary programs for chronic diseases, many patients do not have the financial support/coverage for allied health services needed in a multidisciplinary pain program [22]. For example, many find that their health insurance will not cover some or all of their chronic pain treatment or that they must face a bewildering array of obstacles to having their treatment covered [11]. Innovative low-cost and effective methods for disseminating self-management techniques to a large proportion of patients are necessary given the increasing burden caused by inactivity and chronic disease [23].

Rehabilitation in chronic disease like chronic pain requires a multidisciplinary approach. Self-management support interventions are becoming more common as a structured way of helping patients learn to better manage their chronic diseases, including chronic pain. Because chronic diseases affect the whole person, patient-centered complementary and integrative medicine therapies that acknowledge patients' roles in their own healing processes have the potential to provide more efficient and comprehensive pain management [24, 25].

There is evidence that patient education for self-management can improve the outcomes in chronic diseases. A report of 10 studies involving 6074 people with various chronic diseases, such as arthritis, depression, and chronic pain revealed that self-management programs led to modest, short-term improvements in pain, disability, fatigue, self-rated health, depression, and quality of life when compared to usual care [7]. Evidence also suggests that self-management programs not only helped improve health status but also reduced hospitalizations. In a controlled trial at community-based sites comparing treatment subjects with wait-list control subjects, patients (*N* = 952) 40 years of age or older with heart disease, lung disease, stroke, or arthritis demonstrated improvements at 6 months in weekly minutes of exercise, frequency of cognitive symptom management, communication with physicians, self-reported health, health distress, fatigue, disability, and social/role activities limitations, demonstrating that an intervention designed specifically to meet the needs of a heterogeneous group of chronic disease patients, including those with comorbid conditions, was feasible and beneficial beyond usual care [26].

Mindfulness based cognitive therapy (MBCT) is a form of Mindfulness Based Stress Reduction (MBSR) that includes information about depression as well as cognitive therapy-based exercises linking thinking and its resulting impact on feeling. MBCT demonstrates how participants can best work with these thoughts and feelings when depression threatens to overwhelm them and how to recognize depressive moods that can bring on negative thought patterns [27]. Mindfulness meditation-based therapies alone are being increasingly used as interventions for psychiatric disorders [28], rheumatoid arthritis [9], substance abuse disorders [29], and chronic pain [30]. A meta-analysis of 65 studies reviewed the outcomes of chronic pain programs and reported a 20% average reduction in pain [5].

The effect of mindfulness training has also been shown for the treatment and relapse prevention of anxiety and depression in a variety of baseline medical conditions [27, 31], such as psoriasis [32], back pain [33], anxiety [34], and brain and immune function [35, 36]. In a systematic review and meta-analysis of six clinical trials, 43% of 593 patients with anxiety and depression had reduced risk of relapse as effective as antidepressants [31]. The National Institute for Care and Health Excellence (NICE) Guidelines for depression recommend mindfulness based cognitive therapy, especially for those who are currently well but have experienced three or more previous episodes of depression [37]. Research suggests that meditation improves cognition with evidence of brief mental training. One study found that 4 days of meditation training can enhance the ability to sustain attention, benefits that have previously been reported with long-term mediators [38].

Although there are effective multidisciplinary treatments available, they are often not easily accessible and designed for patients with severe long-lasting problems who are able to cover the costs of these programs. However, the field of Internet interventions is growing [39], and there has been discussion about how the Internet can help as a secondary prevention of chronic pain [40] and can create accessible interventions to reduce risk factors for the development of long-term disability [40].

Over the past decade, researchers worldwide have been using the Internet for online treatment programs, usually behavioral [41–46]. The results of a pilot study assessing the effectiveness of an Internet self-program suggest that a self-management program delivered using an Internet format can lead to statistically significant changes in health efficacy and management of care, fatigue, and depression [47]. Individually tailored Internet-based chronic pain management has shown promising effects on pain at one and 6 months after treatment and QOL at six months in 645 participants [48]. Another study found that while psychological therapies delivered via the Internet reduced pain, disability, depression, and anxiety after treatment, there remains considerable uncertainty around the estimates of effect [49].

Internet-based cognitive behavioral therapy serves as a complement for those with chronic pain who prefer this treatment and have difficulties accessing specialty treatment facilities. In one such study that assessed whether Internet-based intervention would have an effect on the symptoms of chronic back pain, the results showed that the treatment had an effect on catastrophizing and quality of life [50]. This supports an earlier study, which showed significant reductions in catastrophizing, increased control over pain, and ability to decrease pain [51].

Whether pain can be managed through the Internet was examined in a systematic review of randomized controlled trials from 1990 to 2010. The studies evaluated the effects of interventions that provided cognitive and behavioral therapy, moderated peer support programs, or clinical visit preparation or follow-up support on 2503 people in pain. Six studies (35.3%) received scores associated with high quality. Most cognitive and behavioral therapy studies showed an improvement in pain (*n* = 7, 77.8%), activity limitation (*n* = 4, 57.1%), and costs associated with treatment (*n* = 3, 100%), whereas effects on depression (*n* = 2, 28.6%) and anxiety (*n* = 2, 50%) were less consistent. There was limited (*n* = 2 from same research group) but promising evidence that Internet-based peer support programs can lead to improvements in pain intensity, activity limitation, health distress, and self-efficacy; there were limited (*n* = 4 from same research group), promising evidence that social networking programs can reduce pain in children and adolescents and insufficient evidence on Internet-based clinical support interventions. Internet-based interventions seem promising for people in pain, but it is still unknown what types of patients benefit most [52]. However, a recent study demonstrated that teaching people simple positive activities can decrease reported levels of bodily pain and that such activities can be administered over the Internet, a potential avenue for broadly disseminating health interventions at relatively low-costs and with high sustainability [53].

In fact, previous findings have shown that people experiencing pain are more likely to engage in online resources, including sharing their pain experiences and remedies for pain on social networking sites. In fact, evidence shows that patients are beginning to rely on the Internet more frequently as a source of health information [54–60] but still want to discuss such information with their health providers [57]. These results highlight the very significant public health potential of carefully designed and administered Internet-delivered pain management programs and indicate that these programs can be successfully administered with several levels of clinical support. Evidence also suggests that combining high-reading level written material with more accessible video material can improve its impact among patients with less education [58].

In the present study of the patients with moderate or sever**e** pain before treatment, 45% reported mild levels of pain after treatment, with a reduction of severe pain from 40% before treatment to 25% after treatment. Our study extends many other Internet-based pain management programs in that we used social media, such as a blog and Twitter, an information communication technology that is still being explored in rehabilitation. A recent systematic review of ten studies considered efficacy of interventions, such as online health social network websites (*n* = 2), research health social network websites (*n* = 3), and multicomponent interventions delivered in part via preexisting popular online social network websites (Facebook: *n* = 4 and Twitter: *n* = 1). The review revealed significant improvements in outcome measures related to health behavior change (effect sizes ranging from −0.05 (95% CI −0.45 to 0.35) to 0.84 (95% CI 0.49–1.19) [61]. Although social media sites are attractive for disseminating public health messages, they remain underused by health care professionals despite their low-cost and wide reach [59].

The impact of brief but ongoing online patient self-management educations on chronic pain patients who cannot access the conventional multidisciplinary pain programs has not been well explored yet and there is more room for investigation in this area. Our current study is an observational study with the inherent limitations of observational studies, compared to double blind controlled clinical trials. The results can be considered as stepping stones for better designed studies that would allow higher level of statistical rigor with better comparison with control groups and exploring the length of the period of effect for positive changes.

There is evidence that brain hard wiring can change with meditation rather quickly through process of neuroplasticity, suggesting that it may serve as an effective adjunct therapy [62–64]. In our observation, the #Zendose (a daily mindfulness short reflection practice) and Mypain.ca were efforts to get patients' brains busy with the self-management education and activities that would change their passive disease-controlled situation to active self-controlled situation. This was an effort to improve outcomes in patients who could not afford multidisciplinary treatments in clinic settings. Our research, although only observational and with the usual limitations, is unique in that it focused on evaluation of possible outcome improvement in a chronic pain patient population using Internet-based self-management who could not access multidisciplinary care otherwise.

Because there was no control group, statistical comparisons were made to an expected improvement. Also, there may have been particular characteristics about the 200 of 515 patients who agreed to participate that made them more receptive to this type of treatment and thus resulted in a bias in the result. A more robust study would randomize participants to either treatment or control, thereby using outcomes from the two groups for the statistical comparison.

## 5. Conclusion

The Internet is changing the way people are experiencing illness and Internet-based self-management patient education may serve as a complement for chronic pain patients who prefer this treatment or have difficulties accessing specialized treatment facilities. This observation, using Twitter and a blog, showed modest improvement in patients' chronic pain, anxiety, depression, and quality of life using Internet-based patient self-management education tools. These results highlight the need for more studies in this area. Further investigations in this area as an accessible treatment modality that would help to remove the barriers to care seem warranted.

## Figures and Tables

**Table 1:** Pain, depression, anxiety, quality of life, and Patient Global Impression of Change Scores.

Rating scale	Before treatment	After treatment	Change	*p* value^*∗*^
Pain (NPRS)				
Severe	40%	25%	15%	0.0184
Moderate + severe	100%	55%	45%	<0.0001
Depression (HDRS)				
Severe	30%	10%	20%	<0.0001
Moderate + severe	100%	50%	50%	<0.0001
Anxiety (HADS)				
Severe	25%	15%	10%	1.000
Moderate + severe	100%	70%	30%	<0.0001
Quality of Life (QOL)				
Ability to function/daily activities	25%	60%	35%	<0.0001
Patient Global Impression of Change (PGIC)				
Change in activity, symptoms, emotions, and overall QOL	N/A	60%	N/A	

^*∗*^Two-sided test for null hypothesis that the change equals 10% using a general *z*-test [21].

## References

[1] Tsang A., Von Korff M., Lee S. (2008). Common chronic pain conditions in developed and developing countries: gender and age differences and comorbidity with depression-anxiety disorders. *The Journal of Pain*.

[2] Froud R., Patterson S., Eldridge S. (2014). A systematic review and meta-synthesis of the impact of low back pain on people's lives. *BMC Musculoskeletal Disorders*.

[3] Gaskin D. J., Richard P. (2012). The economic costs of pain in the United States. *Journal of Pain*.

[4] Schopflocher D., Taenzer P., Jovey R. (2011). The prevalence of chronic pain in Canada. *Pain Research and Management*.

[5] Flor H., Fydrich T., Turk D. C. (1992). Efficacy of multidisciplinary pain treatment centers: a meta-analytic review. *Pain*.

[6] Lorig K. R., Sobel D. S., Stewart A. L. (1999). Evidence suggesting that a chronic disease self-management program can improve health status while reducing hospitalization: a randomized trial. *Medical Care*.

[7] Franek J. (2013). Self-management support interventions for persons with chronic disease: an evidence-based analysis. *Ontario Health Technology Assessment Series*.

[8] Iversen M. D., Hammond A., Betteridge N. (2010). Self-management of rheumatic diseases: state of the art and future perspectives. *Annals of the Rheumatic Diseases*.

[9] Vermaak V., Briffa N. K., Langlands B., Inderjeeth C., McQuade J. (2015). Evaluation of a disease specific rheumatoid arthritis self-management education program, a single group repeated measures study. *BMC Musculoskeletal Disorders*.

[10] Von Korff M., Gruman J., Schaefer J., Curry S. J., Wagner E. H. (1997). Collaborative management of chronic illness. *Annals of Internal Medicine*.

[11] Jerant A. F., von Friederichs-Fitzwater M. M., Moore M. (2005). Patients' perceived barriers to active self-management of chronic conditions. *Patient Education and Counseling*.

[12] Bjelland I., Dahl A. A., Haug T. T., Neckelmann D. (2002). The validity of the Hospital Anxiety and Depression Scale: an updated literature review. *Journal of Psychosomatic Research*.

[13] Burckhardt C. S., Anderson K. L. (2003). The Quality of Life Scale (QOLS): reliability, validity, and utilization. *Health and Quality of Life Outcomes*.

[14] Ferreira-Valente M. A., Pais-Ribeiro J. L., Jensen M. P. (2011). Validity of four pain intensity rating scales. *Pain*.

[15] McIntyre R. S., Konarski J. Z., Mancini D. A. (2005). Measuring the severity of depression and remission in primary care: validation of the HAMD-7 scale. *Canadian Medical Association Journal*.

[16] Flanagan J. C. (1978). A research approach to improving our quality of life. *American Psychologist*.

[17] Hamilton M. (1960). A rating scale for depression. *Journal of Neurology, Neurosurgery, and Psychiatry*.

[18] Zigmond A. S., Snaith R. P. (1983). The hospital anxiety and depression scale. *Acta Psychiatrica Scandinavica*.

[19] Hurst H., Bolton J. (2004). Assessing the clinical significance of change scores recorded on subjective outcome measures. *Journal of Manipulative and Physiological Therapeutics*.

[20] Scott W., McCracken L. M. (2015). Patients' impression of change following treatment for chronic pain: global, specific, a single dimension, or many?. *The Journal of Pain*.

[21] Altman D. B. (1991). *Practical Statistics for Medical Research*.

[23] Davies C. A., Spence J. C., Vandelanotte C., Caperchione C. M., Mummery W. K. (2012). Meta-analysis of internet-delivered interventions to increase physical activity levels. *The International Journal of Behavioral Nutrition and Physical Activity*.

[24] Delgado R., York A., Lee C. (2014). Assessing the quality, efficacy, and effectiveness of the current evidence base of active self-care complementary and integrative medicine therapies for the management of chronic pain: a rapid evidence assessment of the literature. *Pain Medicine*.

[25] Fu Y., McNichol E., Marczewski K., Closs S. J. (2015). Patient-professional partnerships and chronic back pain self-management: a qualitative systematic review and synthesis. *Health & Social Care in the Community*.

[26] Lorig K. R., Holman H. R. (2003). Self-management education: history, definition, outcomes, and mechanisms. *Annals of Behavioral Medicine*.

[27] Hofmann S. G., Sawyer A. T., Witt A. A., Oh D. (2010). The effect of mindfulness-based therapy on anxiety and depression: a meta-analytic review. *Journal of Consulting and Clinical Psychology*.

[28] Marchand W. R. (2013). Mindfulness meditation practices as adjunctive treatments for psychiatric disorders. *Psychiatric Clinics of North America*.

[29] Brewer J. A., Bowen S., Smith J. T., Marlatt G. A., Potenza M. N. (2010). Mindfulness-based treatments for co-occurring depression and substance use disorders: what can we learn from the brain?. *Addiction*.

[30] Peilot B., Andréll P., Samuelsson A., Mannheimer C., Frodi A., Sundler A. J. (2014). Time to gain trust and change-experiences of attachment and mindfulness-based cognitive therapy among patients with chronic pain and psychiatric co-morbidity. *International Journal of Qualitative Studies on Health and Well-being*.

[31] Piet J., Würtzen H., Zachariae R. (2012). The effect of mindfulness-based therapy on symptoms of anxiety and depression in adult cancer patients and survivors: a systematic review and meta-analysis. *Journal of Consulting and Clinical Psychology*.

[32] Kabat-Zinn J., Wheeler E., Light T. (1998). Influence of a mindfulness meditation-based stress reduction intervention on rates of skin clearing in patients with moderate to severe psoriasis undergoing phototherapy (UVB) and photochemotherapy (PUVA). *Psychosomatic Medicine*.

[33] Cassidy E. L., Atherton R. J., Robertson N., Walsh D. A., Gillett R. (2012). Mindfulness, functioning and catastrophizing after multidisciplinary pain management for chronic low back pain. *Pain*.

[34] Smith B., Metzker K., Waite R., Gerrity P. (2015). Short-form mindfulness-based stress reduction reduces anxiety and improves health-related quality of life in an inner-city population. *Holistic Nursing Practice*.

[35] Davidson R. J., Kabat-Zinn J., Schumacher J. (2003). Alterations in brain and immune function produced by mindfulness meditation. *Psychosomatic Medicine*.

[36] Moynihan J. A., Chapman B. P., Klorman R. (2013). Mindfulness-based stress reduction for older adults: effects on executive function, frontal alpha asymmetry and immune function. *Neuropsychobiology*.

[37] Hopkins K., Crosland P., Elliott N., Bewley S. (2015). Diagnosis and management of depression in children and young people: summary of updated NICE guidance. *British Medical Journal*.

[38] Zeidan F., Johnson S. K., Diamond B. J., David Z., Goolkasian P. (2010). Mindfulness meditation improves cognition: evidence of brief mental training. *Consciousness and Cognition*.

[39] Proudfoot J., Klein B., Barak A. (2011). Establishing guidelines for executing and reporting internet intervention research. *Cognitive Behaviour Therapy*.

[40] Nieto R. (2014). Secondary prevention of chronic pain: can internet help?. *Pain Management*.

[41] Dobson F., Hinman R. S., French S. (2014). Internet-mediated physiotherapy and pain coping skills training for people with persistent knee pain (IMPACT—knee pain): a randomised controlled trial protocol. *BMC Musculoskeletal Disorders*.

[42] Griffiths K. M., Farrer L., Christensen H. (2010). The efficacy of internet interventions for depression and anxiety disorders: a review of randomised controlled trials. *Medical Journal of Australia*.

[43] Marks I. M., Gega L. (2009). Review by Jeroen Ruwaard and Alfred Lange (cognitive behaviour therapy, 2009, 38(2), p. 132) of hands-on-help: computer-aided psychotherapy (maudsley monograph 49) by I. M. Marks, K. Cavanagh, and L. Gega. New York: Psychology Press 2007. *Cognitive Behaviour Therapy*.

[44] Murray E., Burns J., See T. S., Lai R., Nazareth I. (2005). Interactive health communication applications for people with chronic disease. *Cochrane Database of Systematic Reviews*.

[45] Stinson J., Wilson R., Gill N., Yamada J., Holt J. (2009). A systematic review of internet-based self-management interventions for youth with health conditions. *Journal of Pediatric Psychology*.

[46] Wantland D. J., Portillo C. J., Holzemer W. L., Slaughter R., McGhee E. M. (2004). The effectiveness of web-based vs. non-web-based interventions: a meta-analysis of behavioral change outcomes. *Journal of Medical Internet Research*.

[47] Poole J. L., Mendelson C., Skipper B., Khanna D. (2014). Taking charge of systemic sclerosis: a pilot study to assess the effectiveness of an internet self-management program. *Arthritis Care and Research*.

[48] Nevedal D. C., Wang C., Oberleitner L., Schwartz S., Williams A. M. (2013). Effects of an individually tailored Web-based chronic pain management program on pain severity, psychological health, and functioning. *Journal of Medical Internet Research*.

[49] Eccleston C., Fisher E., Craig L., Duggan G. B., Rosser B. A., Keogh E. (2014). Psychological therapies (internet-delivered) for the management of chronic pain in adults. *Cochrane Database of Systematic Reviews*.

[50] Buhrman M., Nilsson-Ihrfelt E., Jannert M., Ström L., Andersson G. (2011). Guided internet-based cognitive behavioural treatment for chronic back pain reduces pain catastrophizing: a randomized controlled trial. *Journal of Rehabilitation Medicine*.

[51] Buhrman M., Fältenhag S., Ström L., Andersson G. (2004). Controlled trial of internet-based treatment with telephone support for chronic back pain. *Pain*.

[52] Bender J. L., Radhakrishnan A., Diorio C., Englesakis M., Jadad A. R. (2011). Can pain be managed through the Internet? A systematic review of randomized controlled trials. *Pain*.

[53] Hausmann L. R. M., Parks A., Youk A. O., Kwoh C. K. (2014). Reduction of bodily pain in response to an online positive activities intervention. *Journal of Pain*.

[54] Ahlwardt K., Heaivilin N., Gibbs J., Page J., Gerbert B., Tsoh J. Y. (2014). Tweeting about pain: comparing self-reported toothache experiences with those of backaches, earaches and headaches. *Journal of the American Dental Association*.

[55] Eysenbach G. (2001). What is e-health?. *Journal of Medical Internet Research*.

[56] Kind T., Huang Z. J., Farr D., Pomerantz K. L. (2005). Internet and computer access and use for health information in an underserved community. *Ambulatory Pediatrics*.

[57] Ruiz I. S., García G. P., Riquelme I. (2014). E-mail communication in pain practice: the importance of being earnest. *Saudi Journal of Anaesthesia*.

[58] Stalker C., Elander J. (2015). Effects of a pain self-management intervention combining written and video elements on health-related quality of life among people with different levels of education. *Journal of Pain Research*.

[59] Vance K., Howe W., Dellavalle R. P. (2009). Social internet sites as a source of public health information. *Dermatologic Clinics*.

[60] Wicks P., Stamford J., Grootenhuis M. A., Haverman L., Ahmed S. (2014). Innovations in e-health. *Quality of Life Research*.

[61] Maher C. A., Lewis L. K., Ferrar K., Marshall S., De Bourdeaudhuij I., Vandelanotte C. (2014). Are health behavior change interventions that use online social networks effective? A systematic review. *Journal of Medical Internet Research*.

[62] Farb N. A. S., Segal Z. V., Mayberg H. (2007). Attending to the present: mindfulness meditation reveals distinct neural modes of self-reference. *Social Cognitive and Affective Neuroscience*.

[63] Nakata H., Sakamoto K., Kakigi R. (2014). Meditation reduces pain-related neural activity in the anterior cingulate cortex, insula, secondary somatosensory cortex, and thalamus. *Frontiers in Psychology*.

[64] Zeidan F., Martucci K. T., Kraft R. A., Gordon N. S., Mchaffie J. G., Coghill R. C. (2011). Brain mechanisms supporting the modulation of pain by mindfulness meditation. *Journal of Neuroscience*.

